# The Impact of High Protein Diets on Cardiovascular Outcomes: A Systematic Review and Meta-Analysis of Prospective Cohort Studies

**DOI:** 10.3390/nu15061372

**Published:** 2023-03-12

**Authors:** Emmanouil Mantzouranis, Eleftheria Kakargia, Fotis Kakargias, George Lazaros, Konstantinos Tsioufis

**Affiliations:** 1 1st Cardiology Clinic, Hippokration Hospital, University of Athens, 115 27 Athens, Greece; 2 Internal Medicine Clinic, 401 General Military Hospital, 115 25 Athens, Greece

**Keywords:** high protein intake, cardiovascular disease, high protein diet, stroke, cardiovascular outcomes

## Abstract

High protein diets have gained increased popularity as a means of losing weight, increasing muscle mass and strength, and improving cardiometabolic parameters. Only a few meta-analyses have addressed their impact on cardiovascular morbidity and mortality and failed to show any significant associations without applying strict values to define high protein intake. Due to the conflicting research background, we conducted a meta-analysis to assess the impact of high protein diets compared to normal protein consumption on cardiovascular outcomes in adults without established cardiovascular disease. Fourteen prospective cohort studies were included. A total of 6 studies, including 221,583 participants, reported data about cardiovascular death, without showing a statistically significant difference in the random effect model (odds ratio: 0.94; confidence interval: 0.60–1.46; *I*^2^ = 98%; *p* = 0.77). Analysis of three studies, which included 90,231 participants showed that a high protein diet was not associated with a lower risk of stroke (odds ratio: 1.02; confidence interval: 0.94–1.10; *I*^2^ = 0%; *p* = 0.66). Regarding the secondary outcome of non-fatal myocardial infarction, stroke, or cardiovascular death, 13 studies that included 525,047 participants showed no statistically significant difference (odds ratio; 0.87; confidence interval: 0.70–1.07; *I*^2^ = 97%; *p* = 0.19). In conclusion, according to our study results, high protein consumption does not affect cardiovascular prognosis.

## 1. Introduction

Cardiovascular disease (CVD) is the leading cause of morbidity and mortality worldwide [1,2]. An unhealthy diet constitutes a major modifiable behavioral risk factor because it exerts a deleterious impact, via long-term effects, on the majority of classic risk factors and metabolic parameters. The interplay between various dietary patterns and CVD has long been investigated under the scope of discovering potential links with hypertension (HTN), dyslipidemia, impaired glucose metabolism and insulin resistance, obesity, and even chronic inflammation and oxidative stress [3,4,5,6,7]. Of note, Ge et al., in a meta-analysis including 21,942 participants, compared the efficacy of 14 popularly named dietary programs, in terms of weight loss and cardiovascular risk reduction, and found that low carbohydrate–high protein (HP) diets, such as the Atkins; low-fat diets, such as the Ornish; moderate macronutrient ones, such as the DASH and Mediterranean, exhibited a significant reduction in weight and blood pressure after 6 months compared to average diets. However, these beneficial effects were largely diminished at 12 months, apart from the persistent LDL reduction attributed to the Mediterranean diet [8].

Protein is a macronutrient that has undergone increasing interest over the last decades. Dietary protein is an integral source of energy in every diet because it provides the essential amino acids for tissue development processes. The role of increased protein intake in cardiovascular health has not yet been elucidated due to the controversial results of clinical studies, especially taking into account the fact that its effects are influenced by quantity, source, and the confounding effects of other macro- and micronutrients.

HP diets, with or without the restriction of carbohydrate intake, have gained increased popularity because they are being promoted as a means of losing weight, increasing muscle mass and strength, preserving a functional skeletomuscular system, improving cardiometabolic parameters, and even cardiovascular outcomes, both in apparently healthy populations and in people at high risk of, or with already established, CVD [9,10,11,12,13,14,15,16,17]. On the other hand, there are studies that appear to associate increased protein consumption with unfavorable metabolic profiles, development, or deterioration of type 2 diabetes mellitus (T2DM) [18,19], worse cardiovascular and renal outcomes [20,21,22], and even with higher all-cause and cause-specific mortality, focusing on cancer and CVD [23,24,25,26]. A U-shaped relationship between protein intake and cardiovascular outcomes is also implied in some studies [27]. Meta-analyses show generally favorable outcomes in regard to weight loss, blood pressure, and metabolic parameters, as surrogate markers to estimate the overall cardiovascular impact [28,29,30,31]. Only a few meta-analyses have addressed hard endpoints and failed to show significant associations between increased protein intake and cardiovascular events, including coronary artery disease (CAD) and stroke. However, they reported superiority for plant protein consumption in cardiovascular prognoses [32,33,34].

A common issue in the aforementioned studies is the vague definition of HP intake. According to a joint expert consultation published by the Food and Agriculture Organization (FAO) of the United Nations, the WHO, and the United Nations University (UNU), the adult population’s recommended dietary allowance (RDA) for protein is considered 0.8 g/kg [35]. Along the same line, dietary recommendations by the European Society for Clinical Nutrition and Metabolism (ESPEN) suggest an RDA of 0.8 g/kg for the general adult population; however, a higher intake is reported as beneficial for the elderly (1.0–1.2 g/kg BW/day) [36]. Of note, the PROT-AGE study group published a consensus document suggesting that protein the RDA is higher than 1.2 g/kg for old adults that engage in exercise training [37]. Recommendations published by the US Institute of Medicine (IOM) [38], embodied in the 2020–2025 Dietary Guidelines for Americans [39], maintain an RDA cut-off value of 0.8 g/kg; however, they define the normal range of daily protein consumption as 10% to 35% of the recommended energy intake, which can be translated to protein consumptions higher than 1.6 g/kg. Focusing on the upper threshold for normal protein consumption the Nordic Nutrition Recommendations suggest up to 20% of the daily energy [40], whereas the Health Council of Netherlands considers 25% as the absolute upper limit [41]. Cut-off values for HP diets in the literature are highly heterogenous and often arbitrary. Based on energy intake, they vary from 15% up to 30% including both clinical studies and meta-analyses [7,10,29,42,43]. Based on grams of protein per kg of body weight (BW), high protein diets seem to range from 1.2 to 1.6 g/kg/day [9].

Interestingly, many important trials and the major meta-analyses designed to assess the cardiovascular impact of dietary protein consumption did not use cut-off values for HP intake, but instead, divided their populations into percentiles according to protein consumption and compared the highest to the lowest. A methodological concern, in this case, is the fact that high percentiles often include people with normal protein (NP) consumption in absolute values, thus, compromising the clinical significance of the results [32,33,34].

Due to the conflicting and ambiguous research background, we decided to conduct a meta-analysis to assess the impact of HP diets compared to NP consumption on cardiovascular morbidity and mortality in adult populations without established CVD.

## 2. Materials and Methods

We conducted a systematic review in accordance with Cochrane guidelines [44]. This meta-analysis was performed according to the 2015 Preferred Reporting Items for Systematic Review and Meta-Analysis (PRISMA) [45]. Our systematic review protocol was registered with the International Prospective Register of Systematic Reviews (PROSPERO).

- Focused questions:
- P (population): patients free of cardiovascular disease.
- I (intervention): high protein diet.
- C (comparison): low or normal protein diet.
- O (outcome): cardiovascular disease.
- Research question: Does a high protein diet affect cardiovascular outcomes?

### 2.1. Search Strategy

The Centre for Review and Dissemination (CRD), Preferred Reporting Items for Systematic Reviews, and Meta-Analyses (PRISMA) and Meta-analysis Of Observational Studies in Epidemiology (MOOSE) guidelines were followed throughout the process of completing this review [46,47,48]. A computer-based search of Medline (PubMed), EMBASE databases, and the Cochrane Library was comprehensively conducted from inception to February 2023. There were no restrictions on the language of the articles. The search term combinations were Medical Subject Heading (MeSH) terms, text words, and word variants for protein diet and cardiovascular disease. The full search strategy and combinations are illustrated in Table 1. The reference lists of all identified articles were hand-searched in case any relevant studies had been missed in the electronic search.

The following search algorithm was used as a basis, and was modified accordingly for each database: (protein diet OR protein intake OR dietary protein consumption OR high-protein score OR carbohydrate diet OR carbohydrate intake OR dietary carbohydrate consumption OR fat intake OR fat diet OR dietary fat consumption OR dietary pattern OR macronutrients intake OR energy consumption) AND (Cardiovascular disease OR CVD OR cardiovascular morbidity OR cardiovascular mortality OR cardiovascular events OR myocardial infarction OR ischemic heart disease OR ischemic heart disease OR coronary artery disease OR coronary heart disease OR stroke OR cerebral infarcts OR intraparenchymal hemorrhage).

### 2.2. Study Selection & Data Extraction

Guidelines suggested using a two-step process for selecting studies for inclusion in a review in order to reduce selection bias. Thus, two reviewers (EK and FK) independently extracted data from the eligible studies using a predesigned extraction form, analyzing each title and abstract independently before meeting to discuss any differences in the selected studies until a consensus was achieved, with the aid of a third researcher (EM). The studies were selected following a review of the titles and abstracts, and then, the full paper was analyzed by the researchers. A study was considered eligible for this meta-analysis if it fulfilled the predefined inclusion criteria: (i) randomized controlled trials and cohort studies with any sample size; (ii) women and men aged at least 18 years old; (iii) mean energy from protein was not less than 18% of the total dietary energy intake in the high protein diet group. Articles meeting one of the following conditions were excluded: (i) men or women who have established cardiovascular disease or chronic kidney disease, (ii) the intervention of the study was not a high protein diet (energy from protein was less than 18% of the total dietary energy intake); (iii) literature with incomplete data. The cut-off value of 18% of the mean energy intake from protein was arbitrarily defined according to international dietary guidelines, in combination with available data from previous meta-analyses and clinical studies. In reality, it represents a daily protein consumption of at least 1.4 g/kg BW for an average adult (men 70 kg, women 60 kg) with an average energy intake of 1800–2300 calories.

When duplicate studies were identified, the most recent study was included, unless the earliest version reported more relevant outcomes. We tried to reduce the risk of ‘double counting’ participants by assessing any same study results reported in multiple articles. Three reviewers (EK, FK, and EM) independently evaluated citations for potential inclusion by screening titles and abstracts and assessed full publications to determine eligibility for final inclusion. A total of 14 studies that met the criteria were, finally, included in the meta-analysis.

Two reviewers (FK and EM) with methodological and content expertise independently extracted information on similar predefined forms relating to the study setting and design, study population, intervention, outcomes, and other relevant information. The extracted information included: Name of the first author, date of publication, location of the study, funding (yes or no), mean age of participants, mean body mass index (BMI) of participants, years of enrollment, years of follow-up, family CVD, hypertension, hypercholesterolemia, diabetes, physical activity, smoking, alcohol consumption, total calories intake per day, protein assessment, total protein intake, protein energy percentage in each arm, primary and secondary outcomes, and results. The main outcomes under investigation were: 1. coronary artery disease (myocardial infarction and revascularization), 2. stroke, and 3. death from cardiovascular causes. Additional outcomes, which were also examined were a composite of the aforementioned. If information relating to the above-mentioned elements was missing, the study’s authors were contacted by e-mail.

### 2.3. Quality and Risk of Bias Assessment

**Figure 1:**
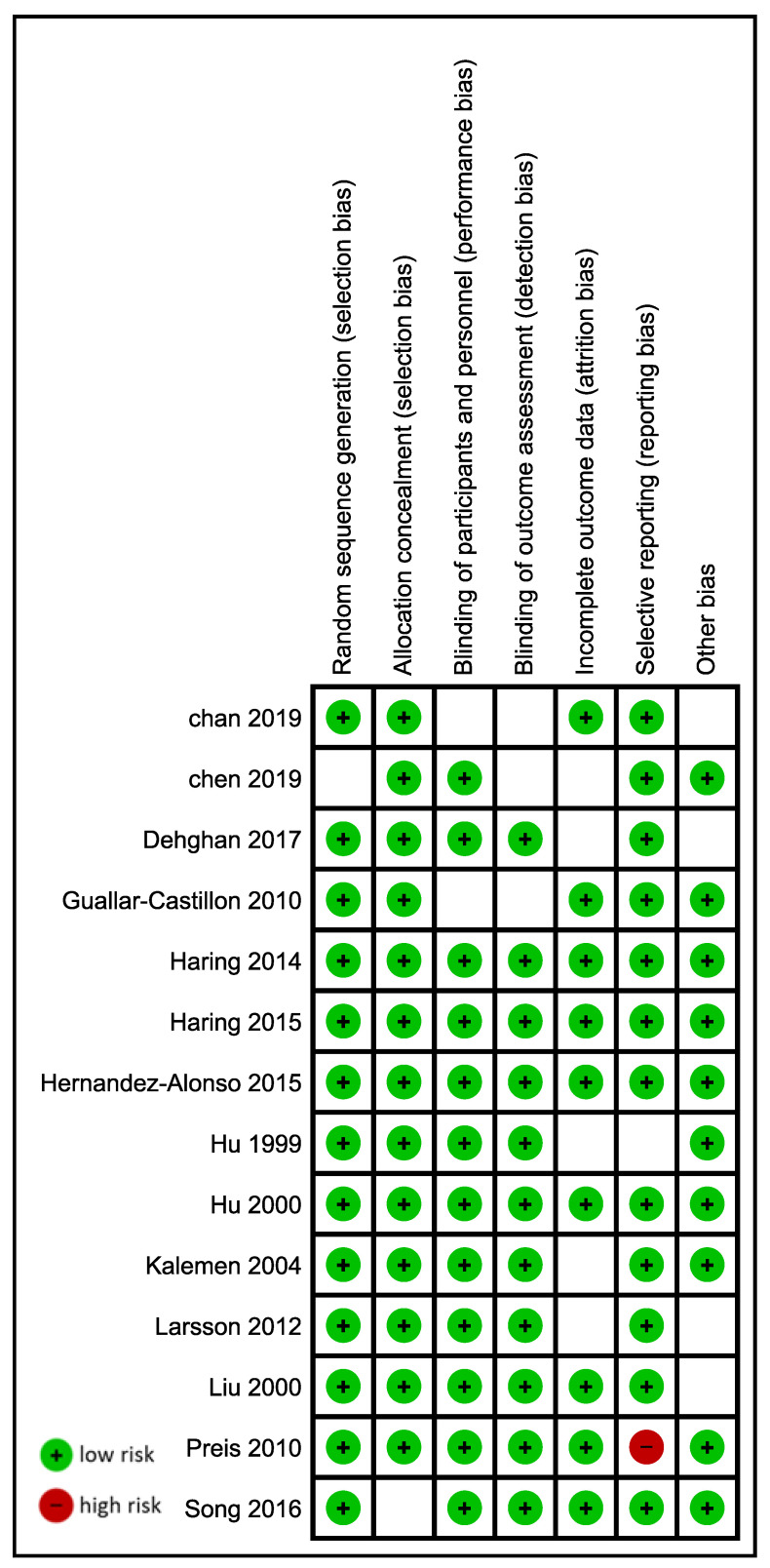
Summary of risk of bias assessment [13,15,23,49,50,51,52,53,54,55,56,57,58].

The methodological quality and risk of bias in each study were evaluated using a study-specific adaptation of the Robins I tool. RoB2 was not used as the included studies were cohort studies. Two reviewers (EM and EK) independently evaluated the risk of bias and rated studies by answering signifying questions on the template, which led to judgments of the low, moderate, serious, and critical overall risk of bias. Disagreement was resolved by a consensus of all authors (Figure 1, Figure S1).

### 2.4. Data Synthesis and Analysis

The statistical analyses were performed using Review Manager version 5.3 software (The Cochrane Collaboration). Confidence intervals (CIs) were set at 95%. Pooled odds ratios (ORs) along with their 95% Cis, for the primary outcomes (cardiovascular death and stroke) and the secondary composite endpoint of cardiovascular morbidity and mortality, were calculated using the random effects model (Der Simonian–Laird). A separate quantitative analysis for myocardial infarction was not conducted as only one study provided data. Interpretation of the results included the study selection strategy, the description of data tabulation, the quantitative analysis of primary outcomes, and the quality assessment of the included studies.

## 3. Results

### 3.1. Search Results

**Figure 2:**
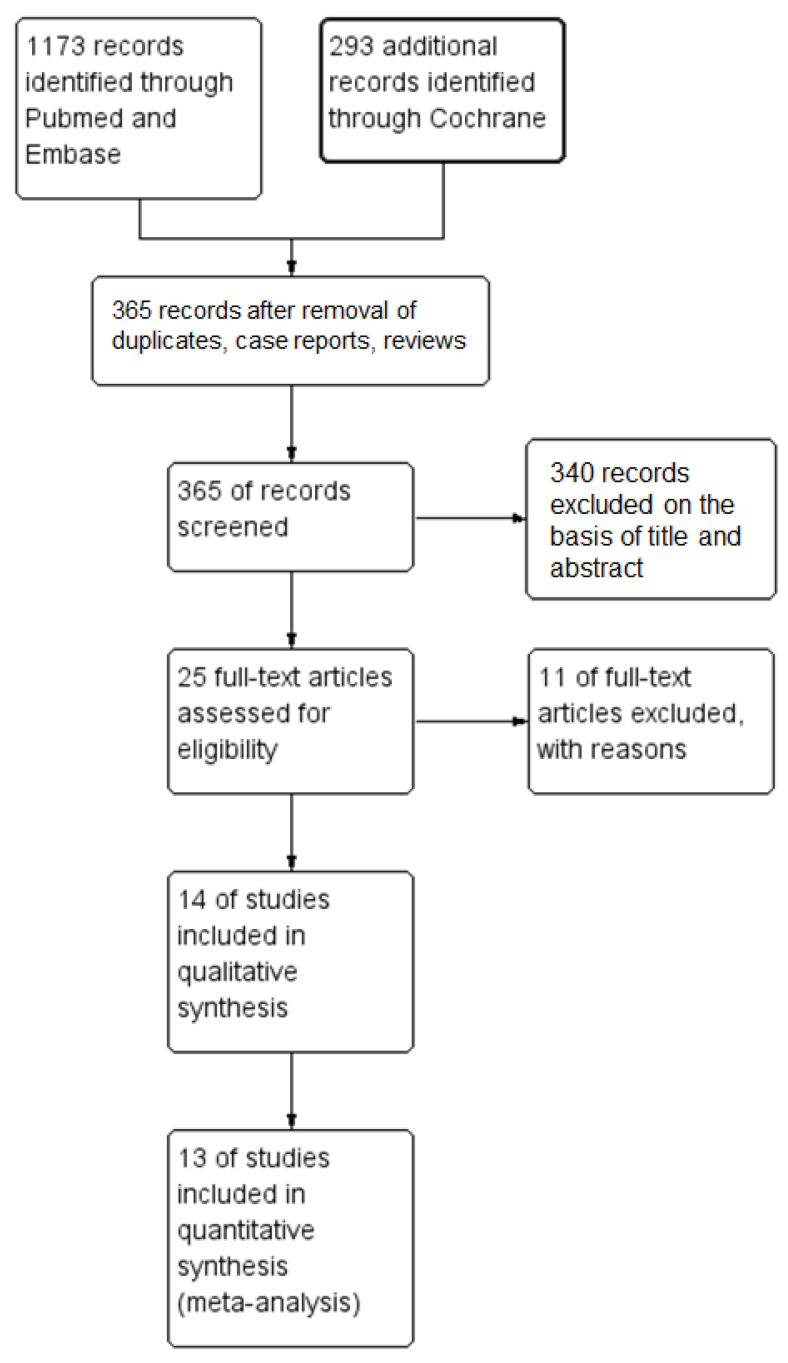
Flow chart of the study eligibility assessment performed according to the Preferred Reporting Items for Systematic Reviews and Meta-Analyses guidelines (PRISMA).

Literature searches yielded 365 articles, after removing the duplicates, case reports, and reviews. After screening the titles and abstracts, 25 relevant articles were retrieved for full-text evaluation. Full-text screening excluded 11 articles (lack of data, exclusion criteria: low protein values and established cardiovascular disease), while 14 studies fulfilled the predetermined eligibility criteria, as shown in the PRISMA flow diagram (Figure 2).

### 3.2. Study Characteristics

A total of 14 prospective studies, including 656,490 participants were included in this meta-analysis. In total, 173,934 participants formed the high protein (HP) group and 329,905, the normal protein (NP) group. Study characteristics and quality assessment are summarized in Table 2. Males comprised 54.07% of the total population, while the mean age and BMI of the study population were 60.4 years old and 26.1 kg/m^2^, respectively. T2DM was moderately prevalent in the study population (7.13% in the HP group and 7.9% in the NP group), while almost 1 in 5 people were reported as active smokers. From the available data, both groups had similar rates of HTN history (24.7% in the HP and 22.6% in the NP group), although a higher prevalence of family history of cardiovascular death was noted in the HP group (41.29% vs. 21.12%). Both groups consumed similar amounts of calories per day (1762 in the HP vs. 1797 in the NP) and mean protein intake (gr/d) was 92.5 in the HP and 72.8 in the NP group. Thirteen of the studies in our systematic review provided enough data to conduct a meta-analysis.

### 3.3. Primary Outcomes

#### 3.3.1. Cardiovascular Death

**Figure 3:**
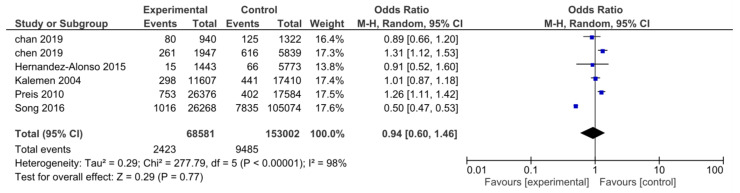
Forest plot for the association between HP intake and risk of cardiovascular mortality [23,25,49,53,55,56,58].

A total of 6 studies, including 221,583 participants, reported data about cardiovascular death. There were 68,581 people in the HP group and 153,002 in the NP group. A total of 11,908 deaths were reported. The analysis showed that there was no statistically significant difference between the two groups in the random effect model concerning cardiovascular death (OR: 0.94; CI: 0.60–1.46; *I*^2^= 98%; *p* = 0.77; Figure 3). The funnel plot was used to evaluate publication bias. The plot was symmetric and did not provide suggestive evidence of publication bias, although the interpretation could have been limited owing to the small number of studies.

#### 3.3.2. Stroke

**Figure 4:**
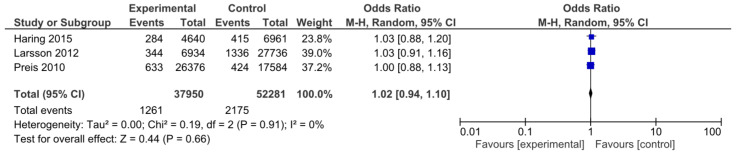
Forest plot for the association between HP intake and risk of non-fatal stroke [15,51,56].

Three studies, which included a total of 90,231 participants, provided data on non-fatal stroke incidence. The HP and NP groups consisted of 37,950 and 52,281 participants, respectively. A total of 3436 strokes were reported. Analysis showed that the HP diet was not associated with a statistically significant lower risk of stroke (OR: 1.02; CI: 0.94–1.10; *I*^2^ = 0%; *p* = 0.66; Figure 4). The funnel plot did not show any publication bias.

### 3.4. Secondary Outcome

**Figure 5:**
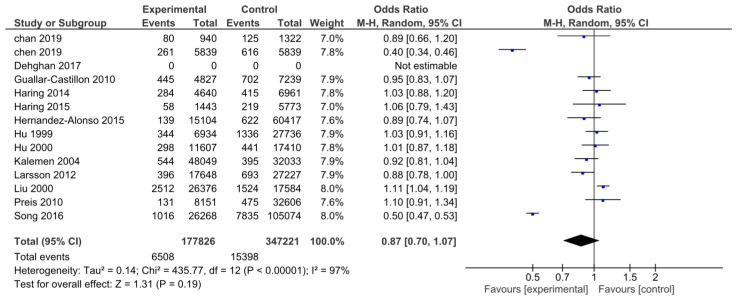
Forest plot for the association between HP intake and the composite endpoint of non-fatal myocardial infarction, non-fatal stroke, and cardiovascular death [13,15,23,25,49,50,51,52,53,54,55,56,57,58].

A total of 13 studies, which included 525,047 participants, provided data on the composite outcomes. There were 177,826 patients in the HP group and 347,221 in the NP group. Overall, 21,906 total cardiovascular events were reported, yet there was no statistically significant difference between the two groups (OR: 0.87; CI: 0.70–1.07; *I*^2^ = 97%; *p* = 0.19; Figure 5). The evaluation of publication bias did not show any bias.

## 4. Discussion

Our study results showed that high protein intake is not associated with an increased risk of stroke, cardiovascular death, and the composite endpoint of all cardiovascular outcomes, including non-fatal stroke, non-fatal MI, and cardiovascular death. Only two studies provided separate data for non-fatal MI, one of them using only incidence rate ratios (IRR) to report events, which made the analysis for this endpoint not applicable. This is the first meta-analysis, to our knowledge, that assessed the impact of “true” HP intake on hard cardiovascular endpoints.

Over the last years, HP diets have been suggested as healthier alternatives that contribute to achieving sustained weight loss or improving muscle mass, while simultaneously preserving good cardiovascular and skeletomuscular health. However, available evidence from cohort studies seems quite controversial, with the majority of studies showing a positive impact of HP diets on cardiometabolic risk reduction, both in apparently healthy populations and in people at a high risk of, or with already established, CVD [9,10,11,12,13,14,15,16,17]. On the other hand, some studies demonstrated a negative impact of HP diets on cardiometabolic profile [18,19] and regarding hard cardiovascular and renal outcomes [20,21,22,23,24,25,26]. Focusing on pathophysiological aspects makes it quite obvious that weight loss, per se, is associated with a better cardiovascular prognosis, and HP diets usually provide a significant weight reduction. Furthermore, adopting HP diets alongside a generally healthier lifestyle warrants the avoidance of excess fat, sugar, and salt, all of which are related to negative effects on cardiometabolic status. On the other hand, the rationale behind HP diets and worse cardiovascular outcomes derives from animal models showing protein-induced atherogenesis. In particular, high levels of circulating amino acids are accompanied by an increase in tissue macrophages, which trigger mTOR signaling to suppress mitophagy. The latter has been related to atherosclerotic plaque progression, through a process involving the accumulation of dysfunctional mitochondria and apoptotic macrophages [59].

Data from previous cohort studies investigating the relationship between protein consumption and cardiovascular prognosis were highly controversial. Interestingly, meta-analyses mainly focused on CVD risk factor reduction by HP dietary patterns showed generally favorable outcomes [28,29,30,31]. However, only three meta-analyses assessed the association between protein consumption and cardiovascular morbidity and mortality. In a recent meta-analysis, including 715,128 participants from 32 prospective cohort studies, Naghshi et al. found that increased protein consumption was related to a lower risk of all-cause mortality, whereas a higher intake of plant protein was related to a reduced risk of all-cause and cardiovascular mortality [34]. Zhang et al., in a meta-analysis of 12 prospective studies, investigated the association between protein intake and stroke incidence in 528,982 participants. No significant association was noticed, however, plant protein seemed to be associated with a reduced risk of stroke [32]. Finally, Qi and Shen, in a meta-analysis composed of 12 prospective cohort studies involving 483,615 participants, showed that higher intake of total protein had no significant association with all-cause cardiovascular and cancer mortality. Regarding the source of protein, increased plant protein consumption was related to reduced all-cause and cardiovascular mortality. On the contrary, animal protein consumption was associated with higher incidences of cardiovascular mortality [33].

The aforementioned studies provided high quality data and showed interesting results and obvious consistency regarding the superiority of plant protein consumption in cardiovascular health. However, there are some methodological concerns that compromise their clinical interpretation. In particular, all three meta-analyses drew their conclusions comparing the highest versus the lowest protein intake category from each study, involving studies that divided their population in percentiles according to protein consumption. This method, although statistically correct, fails to address the real clinical question. That is the long-term cardiovascular footprint of adopting a high protein diet versus a dietary pattern with normal or relatively low protein. Another important concern is the heterogeneity of protein intake values at the highest percentiles among the included studies. Although they might depict the highest protein intake in each respective study population, they often include participants with normal protein consumption values, whereas, in many cases, they fall largely below values considered as high protein intake by any definition used in the literature [16,60,61,62].

Apart from the amount of protein intake, the source of protein has emerged as another major topic of interest. Available evidence from large cohort studies and meta-analyses showed a significant superiority of vegetable protein consumption regarding the improvement of cardiometabolic parameters and the reduced risk of cardiovascular morbidity and mortality. A high intake of plant protein has been associated with reduced blood pressure [43] and a much more favorable lipidemic profile, as shown in a major meta-analysis of 112 randomized clinical trials, conducted by Li et al. [63]. Furthermore, sub-analysis of plant protein intake in the majority of the studies and meta-analyses, addressing cardiovascular morbidity and mortality, has provided much better outcomes compared to animal or total protein intake [23,32,33,34,58,60,64]. Interestingly, in line with these findings, a recent meta-analysis by Glenn et al. showed that vegetarian dietary patterns could be related to reduced CHD incidence and mortality [65]. Despite the evidently favorable results of high plant protein consumption in cardiovascular health, we decided not to perform a separate sub-analysis on that aspect. Our meta-analysis was clinically oriented, and it is common knowledge that no sustainable HP diet is based primarily on vegetables. Animal and dairy protein products are integral parts of human alimentation and their combination with plant sources of protein warrants higher chances of compliance with the dietary pattern. Other sources of dietary protein, such as soy protein [66], have shown beneficial cardiovascular effects, whereas pharmaceutical protein supplements (whey and casein) have long been used as alternative or supplemental solutions to achieve higher daily amounts of protein consumption, usually in the setting of intense physical exercise and muscle growth; however, the aims did not include these parameters in our analysis [67].

A major strength of our meta-analysis is the inclusion of only prospective cohort studies, thus, diminishing the effect of recall and selection biases. Secondly, our analysis sample consisted of 13 prospective cohort studies with a total of 521,155 participants. Reported events included 2548 deaths, 3436 strokes, and 21,906 total cardiovascular events as our composite endpoint and provided enough statistical power to draw conclusions in this study. Furthermore, we excluded studies that included patients who already possessed CVD. Another strong point of our study was in the definition of the composite cardiovascular endpoint as a secondary outcome, because this included all the events of non-fatal stroke, non-fatal MI, and cardiovascular deaths. The novelty of our meta-analysis is that we did not compare extreme percentiles of protein consumption, yet, on the contrary, we made an effort to adhere to a cut-off value of high protein and compared it with normal protein intake, providing a true clinical orientation to our study. Finally, the quality assessment of the included studies demonstrated a low risk of bias.

Our study, however, had some limitations. Most importantly, our total population included both, apparently, healthy people and participants with a variety of cardiovascular risk factors, such as T2DM, HTN, smoking, and a family history of CVD, which was not always reported in the respective studies and could potentially have been important confounders to CVD morbidity and mortality, especially considering the long-term follow-ups. Focusing on the latter, it represents another important limitation, as we included studies with high heterogeneity in the total length of the follow-up time, varying from 4.8 to 32 years. However, most of them reassessed the dietary compliance of participants on a regular basis. Furthermore, the results should be interpreted with the understanding that there was no control for the potential covariates, such as BMI, total energy intake, the intake of other macronutrients, and the effect of physical activity. Of note, all quantitative nutritional parameters were derived from self-reported data, which could have led to over- or underestimating important values, such as protein intake. Unfortunately, there were a few highly relevant studies that could not be included in our analysis due to a lack of reported data. We made unsuccessful attempts to contact the authors. Finally, it must be underlined that the purpose of our meta-analysis was to compare HP diets with NP consumption, in regard to their cardiovascular impact. Most relevant studies, however, used a model of dividing the population into percentiles, according to mean protein intake instead of using a cut-off value. This forced us to select, as the HP intake group, the percentiles with a mean reported protein intake that was higher than 18%, which we quite arbitrarily defined after considering dietary guidelines and previous meta-analyses and studies, as is explained in the methods section.

## 5. Conclusions

Our study results demonstrate that high protein intake is not associated with an increased risk of stroke, cardiovascular death, and the composite endpoint of all cardiovascular outcomes, including non-fatal stroke, non-fatal myocardial infarction, and cardiovascular death, in apparently healthy adults. Results should always be interpreted while considering that existent risk factors and the number of other macronutrients are potent covariates. Further research is warranted to extrapolate these results in populations where chronic conditions are already established.

## Figures and Tables

**Table 1:** Full search strategy.

Group A	*and*	Group B
Protein diet OR		Cardiovascular disease OR
Protein intake OR		CVD OR
Dietary protein consumption OR		Cardiovascular morbidity OR
High protein score OR		Cardiovascular mortality OR
Carbohydrate diet OR		Cardiovascular events OR
Carbohydrate intake OR		Myocardial infarction OR
Dietary carbohydrate consumption OR		Ischemic heart disease OR
Fat intake OR		Ischemic heart disease
Fat diet OR		Coronary artery disease OR
Dietary fat consumption OR		Coronary heart disease OR
Dietary pattern OR		Stroke OR
Macronutrients intake OR		Cerebral infarcts OR
Energy consumption		Intraparenchymal hemorrhage

**Table 2:** Study characteristics and quality assessment.

Study	Country	Type of Study	Population	Years of Enrollment	Age (y)	Follow-Up (y)	Exposure	Exposure Assessment	Exposure Follow-Up	Outcomes
Chan et al., 2019 [49]	China	Prospective cohort	2262 men and women	2001–2003	65 and over	13.8	Quantity and source of protein intake	280-item FFQ	Every 1 year	Death from all causes, cancer, and CVD
Chen et al., 2019 [23]	Netherlands	Prospective cohort	7786 men and women	1989–2008	63.7 ± 8.7	13 (8.3–19.1)	Quantity and source of protein intake	170-item FFQ, 389-item FFQ	Every 3–5 years	All-cause and cause-specific mortality
Dehghan et al., 2017 [50]	International	Prospective cohort	135,335 men and women	2003–2013	50.3 ± 10	7.4 (5.3–9.3)	Dietary intake of fats and carbohydrates	FFQ	Every 3 years	Total mortality and major cardiovascular events (fatal CVD, non-fatal MI, stroke, and heart failure), MI, stroke, CVD mortality, and non-CVD mortality.
Haring et al., 2015 [51]	US	Prospective cohort	11,601 men and women	1987–1989	45–64	22.7	Dietary protein intake	66-item FFQ	6 years from baseline	Non-fatal stroke
Hernández-Alonso et al., 2015 [25]	Spain	Prospective cohort	7216 men and women	2003–2009	men 55–80 women 60–80	4.8	Quantity and source of protein intake	137-item FFQ		Cardiovascular events (i.e., MI, stroke, or death from cardiovascular causes), and death by cardiovascular, cancer, and all-cause
Liu et al., 2000 [52]	US	Prospective cohort	75,521 women	1984	38–63	10	dietary glycemic load, carbohydrate content, and frequency of intake of individual foods	126-item FFQ	every 2 years	Fatal CHD and nonfatal MI
Larsson et al., 2012 [15]	Sweeden	Prospective cohort	34,670 women	1997	61.4 (49–83)	10.4	Quantity and source of protein intake	96-item FFQ		Non-fatal stroke
Kelemen et al., 2004 [53]	US	Prospective cohort	29,017 women	1986	75.8	15	Quantity and source of protein intake	FFQ	At 2, 5, and 7 years	Mortality from all causes, cancer, and CVD
Hu et al., 1999 [13]	US	Prospective cohort	80,082 women	1976	45.8 (34–59)	14	Quantity and source of protein intake	61-item FFQ, 116-item FFQ	Every 2 years	Fatal CHD and nonfatal MI
Hu et al., 2000 [54]	US	Prospective cohort	44,875 men	1986	53.8 (40–75)	8	Prudent and Western dietary pattern score	131-FFQ	Every 2 years	Fatal CHD and nonfatal MI
Preis et al., 2010 (2 studies) [55,56]	US	Prospective cohort	43,960 men	1986	40–75	18	Quantity and source of protein intake	131-FFQ	Every 4 years	Fatal CHD and nonfatal MI, non-fatal stroke
Guallar-Castillon et al., 2010 [57]	Spain	Prospective cohort	40,757 men and women	1992–1996	29–69	11	Westernized and Evolved Mediterranean dietary pattern score	computerized dietary history		CHD events (fatal and non-fatal acute MI or angina requiring revascularization).
Song 2016 [58]	US	Prospective cohort	131,342 men and women	1976–1986	30–75	32	Animal versus plant protein	FFQ	Every 4 years	All-cause and cause-specific mortality

FFQ: food frequency questionnaire; CVD: cardiovascular disease; MI: myocardial infarction; CHD: coronary heart disease. All studies were assessed as high quality.

## Data Availability

Data are available from the corresponding author on the basis of reasonable request.

## Supplementary Materials

The following supporting information can be downloaded at: https://www.mdpi.com/article/10.3390/nu15061372/s1, Supplemental Figure S1: Risk of bias graph; Supplemental Document: PRISMA checklist.

## Abbreviations

CVD: cardiovascular disease; HTN: hypertension; HP: high protein; DASH: Dietary Approaches to Stop Hypertension clinical trial; LDL: low-density lipoprotein; T2DM: type 2 diabetes mellitus; CAD: coronary artery disease; WHO: world health organization; FAO: Food and Agriculture Organization of the United Nations; IOM: US Institute of Medicine; UNU: United Nations University; RDA: recommended dietary allowance; ESPEN: European Society for Clinical Nutrition and metabolism; BW: body weight; NP: normal protein; CRD: Centre for Review and Dissemination; PRISMA: Preferred Reporting Items for Systematic Review and Meta-Analysis; MOOSE: Meta-analysis Of Observational Studies in Epidemiology; MeSH: Medical Subject Heading; PROSPERO: International Prospective Register of Systematic Reviews; BMI: body mass index; FFQ: food frequency questionnaire; MI: myocardial infarction; CHD: coronary heart disease; IRR: incidence rate ratio; OR: odds ratio; CI: confidence interval.
