# Supplementary figures and images for: The Impact of High Protein Diets on Cardiovascular Outcomes: A Systematic Review and Meta-Analysis of Prospective Cohort Studies
Source: Nutrients. 2023 Mar 12;15(6):1372. doi: 10.3390/nu15061372 (PMC10058321; doi:10.3390/nu15061372)

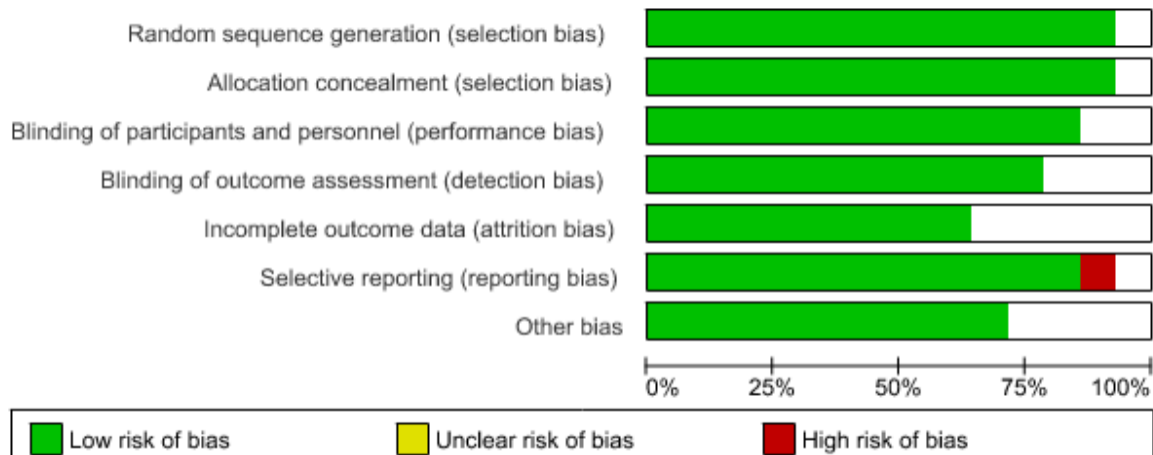

Supplement: Supplementary file 1 [file nutrients-15-01372-s001.zip › nutrients-2257049-supplementary.pdf]
